# Why Do Primary Care Patients Change Their Physicians: An Overview of the Literature

**DOI:** 10.3390/ijerph22020285

**Published:** 2025-02-14

**Authors:** Mariana Cardoso Ribeiro, Elisa Martins, Filipe Prazeres

**Affiliations:** 1 Family Health Unit Beira Ria, 3830-596 Gafanha da Nazaré, Portugal; filipeprazeresmd@gmail.com; 2 Family Health Unit Costa de Prata, 3830-193 Ílhavo, Portugal; elisamaria.180@gmail.com; 3 Faculty of Health Sciences, University of Beira Interior, 6200-506 Covilhã, Portugal; 4 CINTESIS@RISE, MEDCIDS, Faculty of Medicine of the University of Porto, 4200-450 Porto, Portugal

**Keywords:** changing doctor, general practitioner, primary care

## Abstract

Primary healthcare has an important role in a patient’s long-term health. While patients in most countries are free to change their family physician, fragmented care leads to higher healthcare costs, more preventable hospitalizations, and an increased likelihood of deviation from clinical best practice. This review aims to identify the main reasons patients change family doctors, summarize the factors influencing these decisions, and highlight areas in healthcare that can be improved to increase patient satisfaction and design better services. An electronic search of the literature was conducted in March 2023 in PubMed and Embase databases for articles in English, French, Portuguese, or Spanish published from 1980 to March 2023. A thematic synthesis approach was applied to the included studies, involving systematic analysis of their findings to identify and categorize analytical themes. Nineteen relevant studies were identified. The following themes were identified as reasons to change family physicians: doctor–patient relationship; consultation schedule and convenience; referrals and positive references; medication and treatment issues; practice management and cost; personal preferences and physician characteristics; and accessibility and distance. The identified themes can serve as valuable targets for developing interventions aimed at enhancing the quality of care provided to patients.

## 1. Introduction

The World Organization of Family Doctors (WONCA) states that “General practitioners/family doctors are specialist physicians trained in the principles of the discipline. They are personal doctors, primarily responsible for the provision of comprehensive and continuing care to every individual seeking medical care irrespective of age, sex and illness” [1]. Personal doctors have an important role in patients’ long-term health. According to the international literature, public health systems that adopt a model involving GPs or family physicians operating at the local level tend to achieve better results in terms of public health outcomes. Moreover, such systems are generally more cost-effective compared with those that place less emphasis on primary care [2]. Patients have an active role in the system; they are involved in the organization of health services and in improving their quality [3]. Special attention should be given to the quality of the relationship between the patient and his/her family doctor, as well as to problems with medical services that the patient considers unsatisfactory [4].

The family physician–patient relationship is built on clear and empathetic communication and reliability [5]. In almost every country, patients are free to choose their family physicians and can change them if they wish [6]. Continued care is the key to general practice, as it enhances trust, patient satisfaction, information sharing, uptake of preventive care, adherence to medical advice, and reduction of socio-economic disparities, ultimately leading to lower mortality rates [7,8]. While patients should have the freedom to change their doctor with the assurance that this decision aligns with their values, needs, and expectations, frequent change is not to be encouraged [9]. Fragmented care by primary care providers leads to higher healthcare costs, more preventable hospitalizations, and an increased likelihood of deviation from clinical best practice [10]. A systematic review by Joo [11] showed a significant association between fragmented care and worse health outcomes for patients with chronic health conditions, including increased emergency care admissions, overuse of diagnostics tests, and higher healthcare costs. Therefore, changing GPs, especially during ongoing treatment, is not recommended [9].

On the other hand, for patients, changing their GP could be a sign of dissatisfaction and be seen as an opportunity to improve the quality of their care. In England, around 9% of patients choose a new general practitioner every year, and around 1% change practices without changing their address [12]. A study by Empel et al. [12] showed the impact that quality and practice characteristics can have on a patient’s decision to leave their GP without changing their address. Patients often perceive changing their GP as difficult or possible only when relocating to a new area [13]. Patients should not be left feeling neglected or undervalued due to poor communication or barriers to accessibility. By addressing these issues, the healthcare system can become more inclusive and patient-centered.

This review aims to identify the main reasons patients change family doctors, summarize the factors influencing these decisions, and highlight areas in healthcare that can be improved to ultimately increase patient satisfaction and design better services.

## 2. Materials and Methods

To achieve a comprehensive understanding of the reasons why primary care patients change their physicians, a thematic synthesis [14] was conducted. Thematic syntheses are valuable for providing insights into experiences and points of view [14]. It is important to note that this synthesis was not intended to represent a systematic review. While efforts were made to conduct a comprehensive search and synthesize the relevant literature, the methodology employed did not adhere to the criteria set forth for systematic reviews. The authors were not driven by the imperative of locating every relevant study. Instead, conceptual saturation [14] was pursued and considered reached when studies began to repeat the same reasons for patients switching doctors.

In March 2023, electronic searches of the literature were conducted in PubMed and Embase databases for articles in English, French, Portuguese, or Spanish published from 1980 onward. The following MeSH terms were used in the search: “physician–patient relations”, “choice behaviour”, “communication” and “patient satisfaction”. The terms “general practice” and “general practitioners” were used to target studies pertinent to the primary care setting. Similar articles were also manually searched. The search was supplemented by screening reference lists and gray literature. Publications from relevant entities and experts in the field were also sought.

Papers that examined the reasons given by primary care users for changing physicians were included. All study designs and types of publication were included. Given that the review objective was to provide a comprehensive overview of the topic, there was no formal assessment of the quality of individual studies.

Titles and abstracts of the results obtained through the database search were screened, and studies that were duplicated or were not relevant to the primary care setting were excluded. Afterward, all potentially relevant titles and abstracts were further screened for inclusion by two primary care physicians. Full-text articles were then analyzed, and a third author resolved any disagreement. During this process, relevant articles cited in references were also progressively included.

One author independently read the included studies, extracted data using a predetermined form, and consulted other team members to resolve ambiguities. The extracted data included author(s), year of the publication, type of publication, country, purpose, data collection, and the stated reasons for changing physicians.

Thematic synthesis was applied to the included studies, involving a systematic analysis of their findings to identify and categorize analytical themes. This process included a comprehensive review of the findings across studies, in order to identify key concepts and recurring themes [15].

Ethical approval was not required for this study as it solely involved the retrieval and synthesis of data from already published studies.

## 3. Results

The final literature search identified 19 studies relevant to the primary care setting. The 19 studies included fourteen original papers, four research letters, and one article. To facilitate the analysis and presentation of the results, Table 1 elaborates the data about the author/year, type of publication, country, purpose, data collection, and results/conclusions of each study. The articles analyzed were published between 1986 and 2021.

During this review, seven themes representing the main patterns and recurring topics found in the data were identified. These themes included the doctor–patient relationship, consultation scheduling and convenience, referrals and positive references, medication and treatment issues, practice management and cost, personal preferences and physician characteristics, and accessibility and distance.

### 3.1. Theme 1: Doctor–Patient Relationship 

Several papers reported an impaired doctor–patient relationship as a reason for patients changing their physician [16,18,20,21,23,25,30]. This included loss of confidence in the doctor [17], the doctor’s lack of interest or rudeness [6,13,17,31], and poor communication [9,27,31].

### 3.2. Theme 2: Consultation Schedule and Convenience 

Incompatibility of a patient’s schedule with the doctor’s schedule [13,19,21,25], long waiting times and difficulty in obtaining a timely appointment [6,13,17,23,26,31], unavailability of the general practitioner [9,22,24], and issues with surgery hours and convenience [18,26] were reasons for patients changing their physician.

### 3.3. Theme 3: Referrals and Positive References 

A few papers mentioned positive references to another doctor [18,21,26] and a lack of referral for specialized treatment [13,31] as reasons to change physicians.

### 3.4. Theme 4: Medication and Treatment Issues 

Problems with prescribing medication [17,21,24], unsatisfactory treatment received [6,9,13,17,22,31], disagreements with clinical decisions [22], and unavailability of medication [26] led patients to change their physician.

### 3.5. Theme 5: Practice Management and Cost 

Some patients considered the lack of continuity of care [13,17], the presence of rude or unhelpful receptionists [9,13,17,20,23,26], the unavailability of home visits [31], and lack of provision of certain services [9] as reasons for changing physician, in addition to issues with cost and insurance [20,23].

### 3.6. Theme 6: Personal Preferences and Physician Characteristics 

Several papers mentioned how some patients wanted to group their entire family with the same doctor [6,17,21,25,30] or had certain preferences about the physician’s characteristics like age, ethnic group, or gender [13,17,18,22,29,30], the doctor’s character, personality, or behavior [6,24], their education level and proficiency [29], specialist skills [18], and job satisfaction [28].

### 3.7. Theme 7: Accessibility and Distance 

Patients changed their family doctors due to the difficulty accessing the doctor or clinic [6], the distance from the patient’s home [13,17,20,23,29], or because of a change of residency [6,16,20,24,31].

## 4. Discussion

To our knowledge, this article is the first to review and synthesize the main reasons patients change family doctors. Patient satisfaction is influenced by multiple factors, which the patient can perceive as useful, effective, and beneficial [6]. Unsurprisingly, this review found multiple reasons why patients change family physicians. Some reasons are non-modifiable or hard to change, like personal preferences, physician characteristics, accessibility, and distance. However, many factors can be improved to offer a better healthcare system to the patient [6].

The first identified theme was the doctor–patient relationship. Primary care and consistent access to health facilities are associated with better health outcomes. Central to this process is the doctor–patient relationship, where the physician acts as the patient’s advocate, with an emphasis on effective interpersonal communication and mutual confidence [5]. According to the literature, a trusting GP–patient relationship enables continuity of care and improves treatment adherence [32,33,34]. Doctors with better communication skills foster better interpersonal connections, making exchanges of information clearer and easier to understand. This approach involves engaging patients in decision-making, which further strengthens their commitment [24,32]. These practices collectively lead to earlier diagnoses, improvement in patients’ understanding of their conditions, better preventive care, and reductions in adverse events, emergency visits, and hospitalizations [5,6,32]. Surveys have frequently shown patients complaining more about the doctor’s ability to communicate well and their character rather than their clinical knowledge [21,32]. However, a study conducted in England showed that poor communication was not a primary reason for patients switching doctors; other aspects of the doctor’s behavior were mentioned like loss of confidence, lack of interest, and rudeness [17]. It can therefore be assumed that rudeness and disrespectful behavior, whether from physicians or other staff members, should be addressed at the institutional level to maintain trust and professionalism within healthcare settings [6].

The majority of study samples in the literature consisted predominantly of women, reflecting the fact that women tend to change doctors more frequently, in line with some of the studies reviewed [24,25,30]. Women are more sensitive to doctors’ communication skills and the impact on relationships and patient satisfaction [24]. These insights highlight the importance of enhancing doctor–patient relationships by focusing on developing soft skills such as communication and behavioral training, starting as early as medical school. Additionally, addressing factors like work–life balance and other contextual influences can further improve these relationships and patient outcomes [6,24].

The second theme frequently mentioned in the reviewed studies was the consultation schedule, including patient convenience. Occasionally, incompatibility between the patient’s schedule and the doctor’s availability can arise, along with issues related to surgery hours [9,13,18,19,21,22,24,25]. Additionally, poor time management by doctors may result in long waiting lines and difficulty in securing timely appointments. Organizationally, health clinics should find ways to mitigate waiting times for patients to obtain appointments with their family doctor and scheduled clinical appointments [23]. Some ways to improve this issue are to reorganize GPs’ workload by delegating, cooperating with social services, computerizing patient information into a network, being adequately staffed, and using regulated admission, a method used with a pre-determined knowledge of how long an appointment takes. The goal is to ensure that the GPs have sufficient time to engage with patients, fostering stronger doctor–patient relationships, enhancing healthcare delivery, and improving overall patient satisfaction [24,26,31].

The third theme identified comprised referrals and positive references. Patients can influence each other, especially family and friends, to change their GP. This is connected to the patients’ satisfaction and confidence with the healthcare system [26]. The lack of referrals for specialized treatment was also one of the reasons for patients switching doctors; however, this could also indicate a communication problem that demonstrates the inability of the doctor to clearly explain health problems to patients [31].

The fourth theme mentioned was that of medication and treatment issues. Molina et al. [22] showed that dissatisfaction with treatment was the main reason for changing physicians. On the other hand, it was the least mentioned reason in the studies by Billinghurst and Whitfield [17], Vasileva and Karcheva [31], and Buja et al. [24]. Patients may value relational and organizational abilities more and technical and clinical expertise less [21,24]. Improving issues such as denial of services involves educating patients. Some healthcare users, influenced by self-taught information from the internet and higher education, may demand inappropriate prescriptions from their general practitioners [22,24]. These patients should be educated about the prescription process, which requires a thorough examination and mutual decision-making. They should also be guided on how to access accurate information and interpret it correctly. Additionally, it is crucial to inform patients of their rights and responsibilities, as well as those of doctors and healthcare organizations [24]. Many of these discrepancies may originate from a poor doctor–patient relationship that could be resolved through effective communication.

The fifth theme identified, covering practice management and costs, showed the importance that patients also place on continuity of care [17]. Continuity of care is an important factor in primary health care and is even more important in patients with chronic health conditions. It is associated with more controlled access to healthcare and reduction in costs [6,9,26,35,36]. Therefore, changing family doctors is not advised, especially during treatment [9]. Žvinakis et al. [6] showed that 10.5% of patients followed their physician when they changed health clinics, reflecting the importance of the doctor–patient relationship and continuity of care. Patients with high morbidity value continuity of care, even in acute visits. However, many patients prioritize easier access for acute issues over continuity of care [35].

The lack of certain services and the unavailability of home visits are also matters for consideration when patients are changing physicians [9,31]. In the study by Vasileva and Karcheva [31], patients from rural areas were more motivated to change if home visits were unavailable.

The cost of healthcare and insurance coverage are also important factors for some patients. A study by Mold et al. [20] conducted in Oklahoma found that urban patients were more likely to switch doctors for insurance-related reasons and due to a broader range of choices. In contrast, rural elderly patients who tended to have longer-standing relationships with their doctors were less likely to switch for cost or insurance reasons, highlighting the value of the doctor–patient relationship [20]. Patients with greater complexity and multimorbidity experience higher healthcare costs, as demonstrated by multiple studies [37,38]. High healthcare costs harm access to care, therefore decreasing treatment adherence and worsening patient outcomes [39]. These patients are primarily managed in primary care, which plays an important part in maintaining quality of care and controlling healthcare costs [37,38]. A cross-sectional study in Jordan revealed that primary care physicians had a moderate level of cost consciousness. Those with a better understanding of healthcare costs were more likely to provide cost-effective treatments, leading to improved patient outcomes [39].

The sixth theme identified was personal preferences and physician characteristics. Many studies have noted preferences for age, gender, country of qualification, and quality of clinical practice when choosing a general practitioner [12,33,40]. Patients tend to prefer doctors who are the same gender and a similar age as themselves [40]. The studies by Topak and Demirci [29] and Molina et al. [22] found a preference for female physicians. This could be because patients feel more comfortable talking about psychosocial problems with someone of the same gender, or because of women’s health programs like monitoring a pregnancy or performing cervical cytology [22]. Another study in rural Iran found that patients trusted male physicians more than females regarding medical and professional prescriptions [41]. In contrast, a study by Lehmann and Weingarten [18] conducted in Israel found that while some patients did not consider a doctor’s gender important when choosing their physician, they prioritized doctors who shared their language and culture, particularly in a patient population with diverse nationalities. Other studies have shown the influence of ethnicity and language on the quality of doctor–patient relationship [41]. In another cross-sectional study conducted by Nuno et al. in the northern region of Portugal [33], patients showed no preference for the gender or age of primary care physicians. Still, male patients showed a stronger preference for a physician of the same gender and those with an age preference showed a predisposition for ages between 35 and 54 years old, reflecting a balance between an experienced and an up-to-date physician, similar to other studies [33,42]. The Portuguese patients preferred a doctor of the same nationality, while this was irrelevant for foreign patients [33]. The character and personality of the GP were also considered when changing physicians. Among the most mentioned characteristics were doctors’ brusque manner and low availability, including not having the time to pay full attention and listen to their patients, consistent with other studies [24].

Some studies have shown the positive impact of good quality medical treatment [40], and doctors’ clinical knowledge may be considered more important than individual characteristics [29]. However, some studies, such as the questionnaires analyzed by Hernández et al. [21] and Buja et al. [24], suggest that patients place more value on their doctor’s character, personality, and relational and organizational skills rather than their medical knowledge.

Burnout is a work-related syndrome that includes emotional exhaustion, depersonalization, and a sense of reduced accomplishment. Various studies have found a high prevalence of burnout among physicians, with rates reaching 50% in the U.S., and this is estimated to be even higher among family physicians [43,44,45]. A systematic review and meta-analysis examining the global prevalence of burnout among GPs showed that the prevalence of high emotional exhaustion, depersonalization, and decreased personal accomplishment were 37%, 28%, and 26%, respectively [45]. Primary care has been considered the safety net of the healthcare system. Primary care physicians often feel that their work is undervalued [44]. They must attend to many patients while frequently being understaffed, which increases their workload and stress. Additionally, they are also regularly seen as less skilled than other specialists, leading to feelings of demoralization and underappreciation [44,45]. Furthermore, there has been an increase in administrative tasks, less time for clinical work, poor work-life balance, and overall dissatisfaction with their careers [43,44].

Physicians’ job satisfaction and burnout can influence healthcare quality. A satisfied physician leads to higher levels of patient satisfaction and a better patient–physician relationship [28]. In a survey by Nørøxe et al. [28], patients who were registered with a GP who was experiencing high levels of depersonalization, low personal accomplishment, or high burnout were more likely to change their GP compared with those registered with a GP who had lower burnout levels. Depersonalization and a sense of reduced accomplishment were strongly correlated with reduced patient-reported satisfaction. A systematic review and meta-analysis by Hodkinson et al. [43] found a strong connection between physician burnout, career disengagement, and suboptimal health care. Depersonalization appeared to have the most significant impact on quality of care and patient satisfaction.

Various solutions could be implemented to reduce physician burnout, both at an individual and institutional level. At the individual level, strategies such as mindfulness, stress management, and small group discussions can help. At the institutional level, improvements in healthcare organizational culture, workload management, and measures to protect mental health can enhance GP health and job satisfaction [43,45].

The seventh and last theme identified covered accessibility and distance. One important aspect of primary care is that it needs to be accessible to the majority of the population in order to be effective. However, in some areas of the world, ensuring this accessibility is particularly challenging. For example, in some countries, reaching health clinics can be difficult [26]. A study in South Africa by Massango-Makgobela et al. [26] found that 6.8 million children had to travel more than 30 min to reach a clinic. In such areas, transport availability and cost are major causes of missed appointments. Many patients value convenience and proximity to either their home or workplace, which increases the likelihood of regular attendance [17,40,41]. It is important to improve access to healthcare, particularly for those in remote areas, or to implement programs where doctors visit distant homes more frequently [41]. Certain factors are beyond the control of healthcare facilities, such as patients changing residency or wanting to follow family members enrolled at a different clinic [6,16]. A cross-sectional descriptive study showed that, unlike in other studies, transportation or distance were not major reasons for people failing to reach clinics. Instead, most patients in that study changed clinics based on recommendations from friends and family. The study concluded that clinics with high quality services and satisfied patients attract more people from outside of the area [26]. This finding is in line with other studies, such as that by Empel et al. [12] which found that fewer patients left a practice without changing their address when clinical quality and accessibility improved, and other research indicating that patients would follow their family physician if they liked the quality of care, despite the distance [46,47].

While the method of thematic synthesis used in this study provides a rich and in-depth exploration of patient experiences and perspectives, it is important to acknowledge some limitations. The emphasis on conceptual saturation prioritizes depth over breadth, ensuring thorough capture of recurring themes, but it may not fully account for unique or less common findings that could offer additional insights. Additionally, this approach does not adhere to the stronger criteria of systematic reviews, which may introduce the potential for selection bias and the omission of relevant studies. The search was also limited to two databases, which while being comprehensive may have excluded relevant interdisciplinary perspectives or studies indexed in other systems.

In future studies, expanding the search to include additional databases and adopting a broader review methodology such as a systematic review could further enhance the richness and diversity of the findings. These steps could help capture a wider range of studies, including those from diverse disciplines and indexing systems, thereby providing an even more comprehensive understanding of the topic. Furthermore, future qualitative research studies on patient choice could benefit from more robust research methods, as this was a common issue regarding the studies analyzed in this review. Additionally, more research could be carried out using the identified reasons to explore additional ways to improve clinical practice and organization.

Overall, this study is important because it is the first to review the reasons why patients change their primary care physicians. This work provides a starting point for future research and offers valuable insights into the topic.

## 5. Conclusions

This review identifies seven domains that explain why patients may switch doctors: the doctor–patient relationship, consultation scheduling and convenience, referrals and positive references, medication and treatment issues, practice management and cost, personal preferences and physician characteristics, and accessibility and distance. These domains can serve as valuable targets for developing interventions aimed at enhancing the quality of care provided to patients.

## Figures and Tables

**Table 1:** Studies identified in the literature search.

Author/Year	Type of Publication	Country	Purpose	Data Collection	Reasons for Changing Physicians
No author identified [9] 1986	Article	England and Wales	Address the difficulties experienced by patients when changing doctors without changing their addresses.	Questionnaire	The primary reason cited by patients for requesting a change of doctor was poor communication. The second most common reason was unsatisfactory treatment. Other reasons given included dissatisfaction with receptionists, difficulty in reaching the surgery, unavailability of the general practitioner, and a lack of certain services.
Salisbury C.J. [16] 1989	Original paper	England	Investigate how people acquire information about their doctors, why they switch to different practices, and the factors that play a role in their selection of a general practitioner.	Postal questionnaire	One of the most common reasons was a change of residence, with 83% of respondents reporting this as their primary reason for changing their doctor. Another reason that some people may need to switch doctors is due to their previous doctor moving, retiring, or passing away. This only accounted for 1% of respondents, however. Some patients may choose to switch practices because the new location is more convenient for them, which was cited by 5% of the sample. Finally, 5% of respondents reported that they were not satisfied with their previous doctor, which prompted them to seek out a new physician.
Billinghurst B. and Whitfield M. [17] 1993	Original paper	England	Survey patients who voluntarily changed their general practitioner without moving, to identify the reasons for leaving their previous doctor.	Postal questionnaire (open question)	Patients had three main reasons for changing their general practitioner: patient requirements (52.1%), difficulties with the practice (35.6%), and problems with the doctor (35.4%). Distance was the most common reason for changing the general practitioner (40.5%). Other patient requirements were less significant, except for a small percentage of patients who wanted a woman doctor (4.3%) or family registration with a single doctor (5.3%). The most common difficulties with the practice were long waits (13.1%), doctor retired/resigned (8.9%), lack of continuity of care (6.3%), and rude/unhelpful receptionists (5.8%). Patients also stated problems with doctors, including lost confidence (21.4%), the doctor’s lack of interest (10.4%) and rudeness (9.8%), and criticized their prescribing (5.4%).
Lehmann S. and Weingarten M.A. [18] 1995	Original paper	Israel	Obtain information from patients about their reasons for requesting a change of doctor.	Questionnaire (including open questions)	Out of all the requests received, 54% were related to clinical management issues such as incompatibility with the GP, general failure of the doctor–patient relationship, or requesting a GP with specialist skills. Meanwhile, 38% of the requests were related to practice organization, including issues with surgery hours and convenience, the gender of the GP, and the GP’s proficiency in the patient’s mother tongue.
Marco C.G. [19] 1996	Original paper	Spain	Analyze the features of doctors whose change was requested and examine the possible motivations and preferences behind such requests.	Computerized data file	Regarding possible reasons for patients requesting a change of doctor, the majority changed the time of their medical appointments, often switching from morning to afternoon appointments.
Gandhi I.G. et al. [13] 1997	Original paper	England	Determine the reasons for patients switching their general practitioner without changing their place of residence.	Qualitive investigation (interviews and letters)	Four main categories were identified: accessibility, attitudes, management issues, and doctor characteristics. Accessibility was the most frequently mentioned category, with comments relating to the process of gaining access to the doctor, such as issues with the appointment system, distance from the patient’s home, or lack of continuity of care. Attitudes reflected comments about the way doctors or staff behaved towards patients, including rudeness or being uninterested. Management issues were related to diagnosis or treatment, including referral to specialists or non-medical caring professions. Doctor characteristics were the least frequently mentioned category, referring to personal characteristics of the doctor, such as age, ethnic group, or gender.
Mold J.W. et al. [20] 2004	Original paper	United States of America	Investigate the reasons that lead older patients to switch their primary care physicians.	Open-ended question concerning change of primary care physician	Reasons can include insurance or cost issues, a move to a new location, or their previous doctor leaving, retiring, or passing away. The distance to the doctor or hospital may also be a factor, along with dissatisfaction with their current doctor. Recommendations from family members, friends, or other doctors may prompt patients to switch physicians. Additionally, problems with the staff at the doctor’s office could contribute to a decision to find a new primary care physician.
Hernández M.L. et al. [21] 2007	Research letter	Spain	Find out why patients change their family doctor voluntarily and compare the reasons given by active and pensioner patients.	Self-administered survey using modified Delphi technique	Patients changed doctors primarily due to poor doctor–patient relationships, with 32% of active patients and 31% of pensioners citing this as a reason. Consultation schedules and positive references to another doctor were the second most common reasons, with 28% of active patients and 27% of pensioners citing these factors, respectively. Other common reasons included difficulty in obtaining temporary disability (20.2% for active patients) and not receiving prescribed medication (16% for pensioners). Additionally, positive references to another doctor (13% for active patients) and grouping the entire family with the same doctor (11.8% for pensioners) were also reasons for switching doctors.
Molina J.T. et al. [22] 2007	Original paper	Spain	Explain the factors that lead patients to change their doctor.	Telephone survey	The primary reason cited for changing doctors was dissatisfaction with the treatment received, accounting for 35.5% of respondents. Disagreements with the physician’s clinical decisions were the reason for change in 22.6% of cases. The remaining 38.7% of respondents cited various other reasons, such as the physician’s limited availability or a preference for being seen by a female doctor.
Wessels J.J. and Viljoen W. [23] 2009	Research letter	South Africa	Identify the primary factors that may lead a patient to leave a general practice.	Qualitative questionnaire	From the sample, 27 patients left the practice due to living too far away; 5 patients had to use the public health system as they could not afford the consultation fees; 10 patients left the practice because they were unhappy with it, citing reasons such as long waiting times, interpersonal problems with the doctor or support staff, and financial difficulties.
Buja A. et al. [24] 2010	Original paper	Italy	Identify the reasons why people choose to change their general practitioners.	Self-administered, structured questionnaire	About one-third of the sample (31%) mentioned personal reasons (e.g., change of residence), while 16% cited reasons related solely to the physician. Most of the sample (53%) cited both types of reasons. The most common reasons were related to the character and personality of the GP, particularly an excessively hasty manner. Scarce availability was the second most frequent reason, followed by denial of services, disorganization, and problems with prescribing drugs.
García-Basteiro A.L. et al. [25] 2013	Research letter	Spain	Investigate why patients change their primary care physician.	Forms for requesting a change of doctor (list of 14 different reasons, from which the main reason for the change must be chosen)	The main reason for patients to request a change of primary care physician was the incompatibility of their schedule with their assigned doctor’s schedule, which was reported in 30.3% of cases. The desire of families to be grouped with the same doctor was the second most common reason, at 18.4%. Interestingly, dissatisfaction with the doctor–patient relationship was a relatively low cause of change, at only 21%.
Masango-Makgobela A.T. et al. [26] 2013	Original paper	South Africa	Investigate the reasons why individuals choose to stop using their nearest healthcare service and instead opt for the Karen Park Clinic in Pretoria North.	Questionnaire	Several reasons were identified for why individuals chose to attend the Karen Park Clinic in Pretoria North instead of their nearest healthcare service. Firstly, many patients were referred to the clinic by friends and family members, and also because of the doctors and nurses working there. The unavailability of medication at their nearest clinic also contributed to their unwillingness to attend, along with long waiting periods and long queues due to large numbers of patients. Finally, some patients reported being deterred from their nearest clinic by rude staff.
Nagraj S. et al. [27] 2013	Original paper	England	Investigate whether rates of voluntary disenrollment in England are associated with poor patient experience, practice and doctor characteristics, or the availability of other family practices in the locality.	Data from the English national GP Patient Survey	Family practices with low levels of patient satisfaction, particularly in terms of doctor–patient communication, were more likely to have high rates of patient disenrollment.
Nørøxe K.B. et al. [28] 2019	Original paper	Denmark	Investigate the relationship between the job satisfaction, well-being, and self-assessed workability of general practitioners (GPs) and their patients’ tendency to change GP.	Nationwide questionnaire survey combined with register data	The likelihood of patients changing their GP was higher if the job satisfaction of their GP was lower. Likewise, patients who were registered with a GP who experienced high levels of depersonalization, low personal accomplishment, or high burnout were more likely to change their GP compared with those registered with a GP who had lower burnout scores.
Topak N.Y. and Demirci H. [29] 2019	Original paper	Turkey	Evaluating the factors that influence patients’ decisions to change their family physician.	Questionnaire	The study identified three primary factors that were reported as the most significant reasons for patients changing their family physician. These factors were the distance to the practice, the doctor’s education level, and the doctor’s gender, accounting for 52.7%, 25.8%, and 16% of the reported reasons, respectively.
Prazeres F. and Passos L. [30] 2020	Research letter	Portugal	To evaluate the reasons given by patients for changing their doctor.	Forms for requesting a change of doctor	The most common reason for changing physicians was based on personal preference (44.4%) such as having a doctor of a specific gender or choosing the same physician as other family members. Dissatisfaction with the doctor–patient relationship (40.7%) was also a common reason for changing physicians.
Vasileva B.I. and Karcheva M.D. [31] 2021	Original paper	Bulgaria	Analyze patients’ motives for changing GP.	Survey containing closed, semi-open, and open-ended questions	The most frequent reason for changing GPs was long waiting time (30.2%), followed by permanent lack of referral to a specialist (11.7%) and change of residence (19.0%). Other reasons for changing GPs included being unsatisfied with the health care received (4.0%), reluctance to explain health problems (6.0%), not responding to home visits (3.5%), experiencing a bad or rude attitude from the GP (2.3%), and not receiving effective treatment (1.2%).
Žvinakis P. et al. [6] 2021	Original paper	Lithuania	Understand why patients move from public to private primary healthcare (PHC) providers and how the reasons may be linked to the patients’ demographic characteristics.	Phone questionnaire	The patients were divided into four groups based on the reasons they provided. The first group (43.4%) cited personal reasons for switching, such as inconvenient location of the public institution department, moving to a new location, or enrolling at the same private PHC provider as family members. The second group (25.2%) cited reasons related exclusively to their family physician, such as treatment failure, insensitivity to the patient’s problems, denial of requested services, or disrespectful behavior. Some patients also enrolled with a former family physician who shifted from a public to a private PHC organization. The third group (24.9%) cited reasons related only to PHC institutions, including long waiting times and queues to obtain a family doctor’s appointment. Finally, the fourth group (8.6%) mentioned two or more reasons related to personal factors, the family physician, and/or PHC institutions.

## Data Availability

The original contributions presented in this study are included in the article. Further inquiries can be directed to the corresponding authors.
